# A case of hepatitis E that developed during chemotherapy for malignant lymphoma and responded to ribavirin

**DOI:** 10.1002/cnr2.1957

**Published:** 2023-12-20

**Authors:** Seiya Hashimoto, Hirofumi Fukuda, Kohei Takeda, Keiichi Uchida, Fumiaki Sanuki, Takashi Akiyama, Eisei Kondo, Hideho Wada

**Affiliations:** ^1^ Department of Hematology Kawasaki Medical School Kurashiki Japan; ^2^ Department of Pathology Kawasaki Medical School Kurashiki Japan

**Keywords:** chemotherapy, hepatitis E virus, ribavirin

## Abstract

**Background:**

The main differences in cases of sudden elevation of hepatic enzyme levels during immunochemotherapy are the reactivation of the hepatitis B virus or drug‐induced liver injury. Here, we report a case of acute liver injury caused by the hepatitis E virus (HEV) during chemotherapy for malignant lymphoma, wherein the patient was successfully treated for the hepatitis and resumed chemotherapy to completion.

**Case:**

A 57‐year‐old woman visited her local doctor because she felt lightweight and tired. The patient underwent lower gastrointestinal endoscopy and was diagnosed with a malignant lymphoma of the small intestine (diffuse large B‐cell lymphoma). The patient had a history of oral consumption of undercooked pork liver to improve anemia and was diagnosed with acute hepatitis E.

**Conclusion:**

This report highlights the successful treatment of HEV infection in a patient undergoing immunosuppressive therapy for malignant lymphomas. A novel aspect of this study is the safe and effective use of ribavirin, an antiviral medication, along with continued chemotherapy, which resulted in sustained virological response (SVR) and the completion of the planned chemotherapy regimen. This report also provides new insights into the management of HEV infections in immunosuppressed patients undergoing chemotherapy and emphasizes the importance of considering HEV as a potential cause of acute liver injury in such cases. The successful use of ribavirin along with continued chemotherapy offers a promising treatment strategy for clinicians to consider in similar scenarios.

## 1 CASE

A 57‐year‐old woman was diagnosed with malignant lymphoma in July 2000 after a positive stool occult blood test was identified during a physical examination, and rituximab (RTX), cyclophosphamide (CPA), doxorubicin (DXR), vincristine (VCR), and prednisolone (PSL) (R‐CHOP: RTX 510 mg, CPA 1030 mg, DXR 65 mg, VCR 1.9 mg, PSL 65 mg) chemotherapy was administered. This chemotherapy was delivered in cycles of 21 days per cycle. All investigation and therapies were performed at Kawasaki Medical School Hospital.

Blood tests revealed abnormal liver enzyme levels during the two courses of chemotherapy. The patient was asymptomatic, no new medications were administered, and there was no significant medical history. Laboratory tests revealed no signs of chronic hepatitis. The results of liver function tests were as follows: aspartate aminotransferase, 395 (10–42 U/L), alanine aminotransferase, 235 (13–30 U/L), alkaline phosphatase, 169 (38–113 U/L), and total bilirubin, less than 0.2 (0.4–1.5 < mg/dL). Hepatic synthesis function was normal. Ultrasonography of the liver was normal, and contrast‐enhanced computed tomography (CT) revealed no organic abnormalities. Before referral to our hospital, the patient had been suffering from anemia due to gastrointestinal bleeding. She received an RBC transfusion at another hospital; therefore, her symptoms may have been caused by a transfusion‐transmitted infection. The patient was screened for hepatitis A, B, and C viruses; human immunodeficiency virus; herpes virus; and cytomegalovirus. The results of all screening tests were negative. In addition, although the patient was of an age at which autoimmune diseases are common, the liver autoantibody test results were normal. Therefore, autoimmune diseases, such as primary biliary cirrhosis and autoimmune hepatitis, were ruled out. Since the patient had a history of eating undercooked pork, we considered the possibility of hepatitis E and measured HEV‐RNA, which was positive at 1.63 × 102 IU/mL.

**FIGURE 1:**
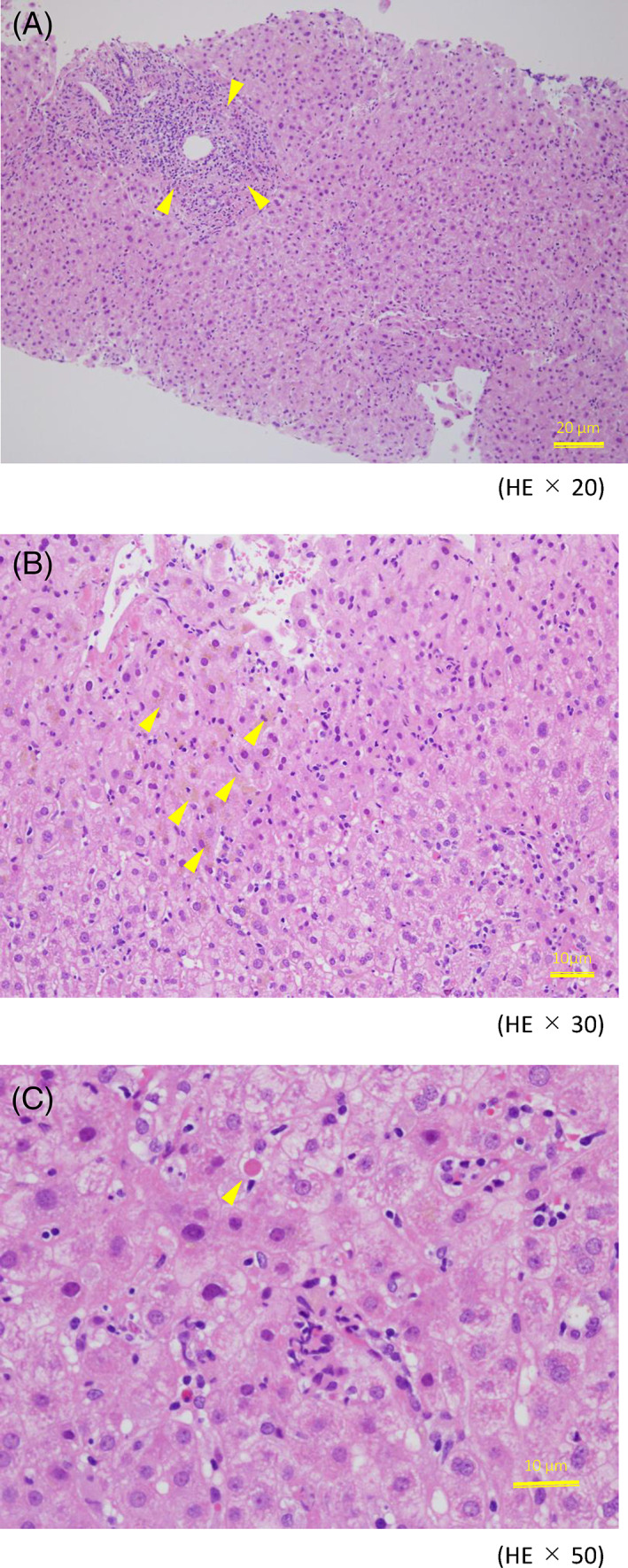
The histological picture of hepatitis E is said to resemble that of hepatitis A. Inflammatory cell infiltration in the portal and periportal regions and bile stagnation in the parenchymal region within the lobule have been reported. In the present case, (A) inflammatory cell infiltration in the portal vein area (yellow arrowheads, A), bile stagnation in the parenchymal region within the lobule (indicated by yellow arrowheads, brown area, B), and the appearance of eosinophils in some lobules (yellow arrowheads, the structures seen after changes due to parenchymal damage, C) were also characteristic findings. The histological picture of acute hepatitis E was considered consistent with acute hepatitis E.

An acute‐phase histological lesions are characteristics of HEV infection.^1^ A liver biopsy in this case revealed that the inflammation was composed mainly of lymphocytes mixed with histiocytes and eosinophils, and its distribution is usually considered to be present in all zones, 1–3, especially zone 3 (Figure 1). Therefore, in this case, although relatively few with zone 3, which is considered typical, inflammatory cell infiltrates were prominent in the portal vein area, whereas some areas in the parenchyma of lobular zone, mainly in zone 1. In some hepatocytes, including those around the central vein, inflammation was relatively mild, and bile pigment deposition was observed, suggesting the possibility of bile stasis. Taken together, we considered this case to be partially consistent with the findings of hepatitis E.

Although there are no pathological findings specific to hepatitis E, some reports suggest that the liver pathology of hepatitis E often presents with acute‐phase histological lesions. Hepatitis E is characterized by biliary stasis of the liver, a pseudotubular arrangement of hepatocytes around dilated small vessels, and proliferation of the bile ducts. Hepatic biliary stasis has been reported in 58% of biopsied livers with histological evidence of biliary stasis and has been observed in many HEV‐infected patients.^2^

Consistent with the findings of hepatitis E in a previous report, there were inflammatory cell infiltrates, including lymphocytes and histiocytes, mainly in zone 1; bile stagnation, including the portal vein area; and coagulation necrosis, including the appearance of eosinophils.

Several studies have reported the initial dose of ribavirin for hepatitis E, with the dose adjusted based on the estimated glomerular filtration rate or weight‐adjusted dose.^3^

**FIGURE 2:**
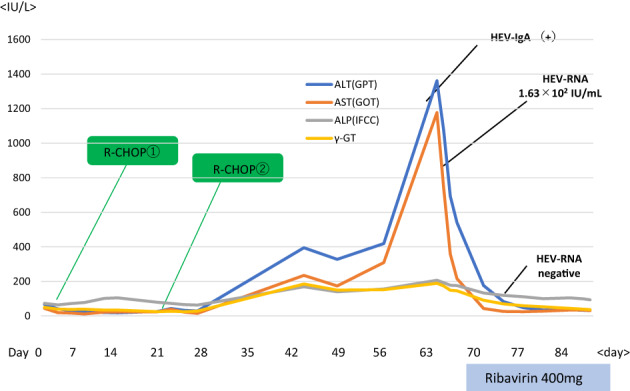
Before the third course of chemotherapy, rapid increase in liver enzymes appeared. A medical interview revealed that hepatitis E virus HEV‐IgA was positive for this patient. The patient was started on oral ribavirin, and the elevated liver enzymes rapidly improved; HEV‐RNA became negative within 2 weeks after the start of the treatment.

**FIGURE 3:**
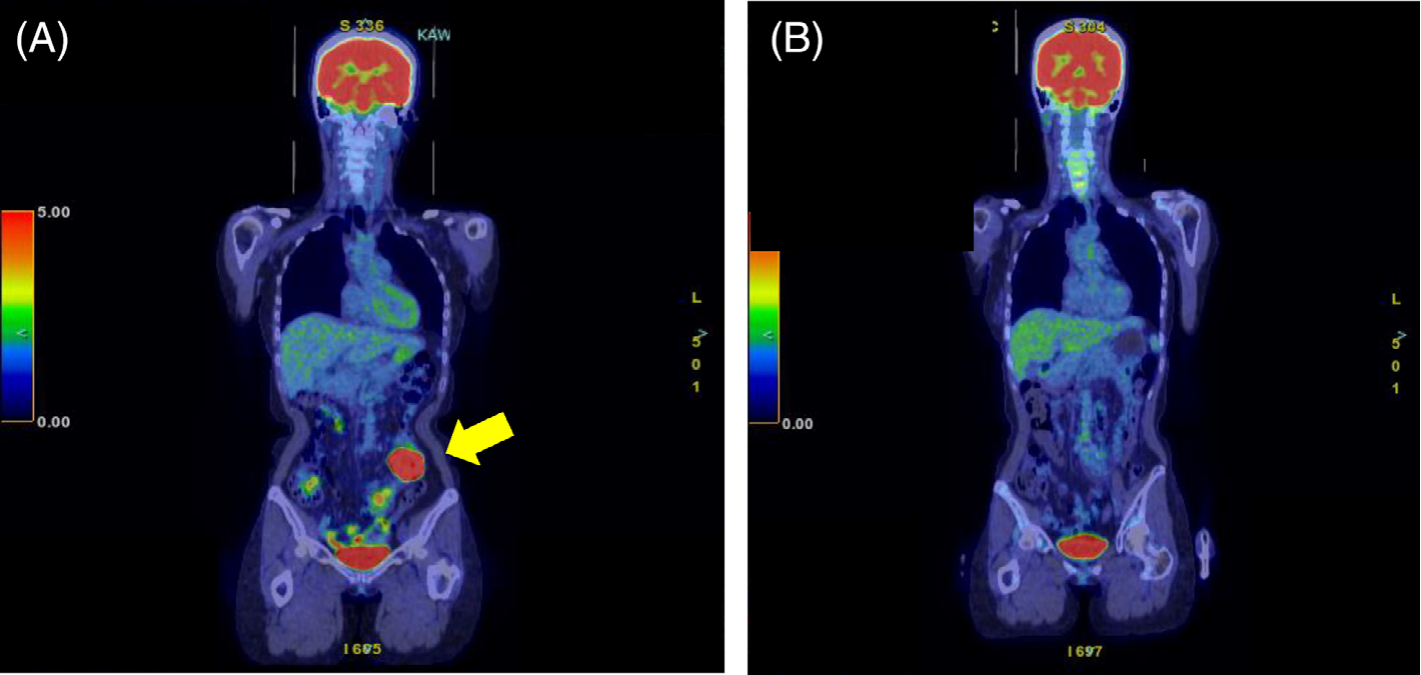
The accumulation of fluorodeoxyglucose in the left lower abdomen (indicated by yellow arrow) seen at the time of initial diagnosis (A) disappeared after 6 courses of chemotherapy and is still in complete remission (B).

The patient was started on 400 mg of ribavirin at a weight‐adjusted dose, and blood tests showed no apparent drug‐related adverse events. After treatment initiation, HEV RNA became almost undetectable (Figure 2); therefore, R‐CHOP therapy was resumed. Positron emission tomography/computed tomography confirmed complete remission after six courses of chemotherapy (Figure 3).

After completing a series of treatments, she was followed up every 2 weeks at an outpatient clinic in our hospital until hepatobiliary enzyme improved within normal limits. And since then, she has been doing well with no recurrence of her lymphoma and no reactivation of HEV at least 2 years.

## 2 DISCUSSION

The first case of hepatitis E transfusion infection in Japan was reported in 2020.^4^ However, cases of hepatitis E infection due to blood transfusion have been confirmed as early as 3 weeks after transfusion. Although this patient also had a history of blood transfusion, we contacted the Japanese Red Cross Society and excluded infections caused by blood transfusion.^5^

The most important finding of this case was that the patient achieved SVR despite receiving chemotherapy. Ghandili et al. reported that patients receiving chemotherapy had a significantly higher viral load of HEV and a high mortality rate of seven out of 20 patients during active chemotherapy. However, the patient in our report was doing well despite receiving chemotherapy without HEV reactivation.^6^

The differential diagnosis in patients with elevated aminotransferase levels undergoing drug treatment requires consideration of various causes. The diagnosis of HEV infection is often difficult, especially because drug toxicity‐related causes must be excluded and symptoms vary considerably from patient to patient.

Generally in these cases, including ours, there is great diversity in the immune status of patients undergoing chemotherapy, and serodiagnosis alone may not accurately detect HEV infections. Therefore, when HEV infection is suspected in immunosuppressed patients under special conditions, HEV polymerase chain reaction (PCR) should be performed simultaneously with serology for an accurate diagnosis. Bettinger et al. reported that seroconversion to anti‐HEV IgM and IgG was observed more than 2 weeks after the initial detection by PCR.^7^ Similar to that report, in this case we were able to accurately diagnose the disease not only serologically but also by HEV‐PCR. The immune status of patients undergoing chemotherapy is very diverse, and it is advisable to perform not only HEV serological diagnosis but also HEV PCR.

In general, the treatment of acute HEV infection is supportive; however, some patients may require the discontinuation of immunosuppressive drugs or ribavirin treatment. These included cases in which prednisolone was administered, as in the present case.^8^

Previous reports have shown that ribavirin, either alone or in combination with peginterferon, is effective for the treatment of chronic HEV in patients with immunocompromised patients including hematological diseases.^9^ However, there are no data on the recommended duration of these treatments, particularly the use of ribavirin. Previous studies have shown that ribavirin therapy may be necessary for high‐risk patients, such as those undergoing chemotherapy for gynecological cancer.^7^

Another case of failure to respond to ribavirin was documented by Miyoshi et al., where the prognosis of that case was different from that of ours because it was a Burkitt type lymphoma, but the major difference is that our case achieved SVR.^10^ Furthermore, it has been reported that HEV is more likely to become chronic in immunosuppressed states, such as in organ transplant patients and hematopoietic stem cell transplant patients.^11^ Although the patient was treated only with chemotherapy, he was also treated with rituximab and other antiviral therapies in an immunosuppressed state, and the risk of chronicity and fulminant hepatitis was considered high; however, the use of ribavirin prevented these risks.

Several reports have shown that immunosuppressed patients are at high risk of HEV infection, which may have serious clinical consequences owing to prolonged HEV autoproliferation. The patient was treated with anti‐CD20 antibodies and cyclophosphamide for hematological tumors. The chronic course of the HEV infection may have been caused by immunosuppression. The first step in the treatment of patients with chronic infections is the reduction or cessation of immunosuppressive drugs^12^; in this case, the patient was started on ribavirin therapy after adjusting the dosage of chemotherapy drugs. The patient safely completed treatment, and complete remission was achieved.

Determining the diagnosis of drug‐induced liver injury may be disadvantageous for the patient because it could be related that the treatment will be able to be continued or not.

One issue for the future is the late inclusion of hepatitis E as a differential diagnosis of elevated liver enzyme levels. Acute HCV and HEV infections are strongly suspected clinically in patients with acute hepatocellular liver injury and suspected drug‐induced liver injury, as in this case.^13^ In addition, similar to HBV hepatitis, CD20‐directed monoclonal antibodies seem to be risk factors for hepatitis E.^6^, ^14^

This case may be considered a significant report that underscores that timely diagnosis and initiation of antiviral therapy are important to ensure optimal outcomes for patients on chemotherapy.

In conclusion, patients with unexplained acute or chronic hepatitis during immunosuppression should be considered for possible HEV infection and tested using PCR. In addition, patients receiving immunosuppressive therapy should be carefully monitored for the chronic course of the HEV. In this study, hepatitis E that developed during immunosuppressive therapy was safely treated with ribavirin, and chemotherapy was continued with complete remission of malignant lymphoma. This study provides clinicians with new information and suggestions for improving the clinical outcomes of patients with HEV infections.

## AUTHOR CONTRIBUTIONS

All authors listed have significantly contributed to the investigation, development, and writing of this article. Hirofumi Fukuda: writing, original draft preparation, writing, review and editing, and supervision.

## CONFLICT OF INTEREST STATEMENT

All authors declare no conflicts of interest.

## ETHICS STATEMENT

Not applicable.

## PATIENT CONSENT STATEMENT

Written informed consent was obtained from the patient.

## Data Availability

Data sharing is not applicable to this article as no new data were created or analyzed in this study.
